# Roflumilast mitigates cartilage degeneration in osteoarthritis by activating CFTR-mediated anti-apoptotic signaling

**DOI:** 10.1186/s13075-026-03872-0

**Published:** 2026-07-24

**Authors:** Haoyu Yang, Guihua Tan, Xunhao Wang, Nuo Xu, Wenli Gong, Ruiyang Jiang, Xiaoyu Jin, Chenfeng Qiao, Zhaofeng Zhang, Gang Zhao, Dongquan Shi, Jingyi Mi

**Affiliations:** 1 Department of Orthopedics, Wuxi Ninth People’s Hospital Affiliated to Soochow University, Wuxi, Jiangsu China; 2 Division of Sports Medicine and Adult Reconstructive Surgery, Department of Orthopedic Surgery, School of Life Sciences, Nanjing Drum Tower Hospital, Nanjing University, 321 Zhongshan Road, Nanjing, Jiangsu 210008 PR China; 3 Suzhou Medical College of Soochow University, Suzhou, China; 4 Division of Sports Medicine and Adult Reconstructive Surgery, Department of Orthopedic Surgery, Nanjing Drum Tower Hospital Clinical College of Nanjing University of Chinese Medicine, 321 Zhongshan Road, Nanjing, Jiangsu 210008 PR China

**Keywords:** Roflumilast, Apoptosis, Oosteoarthritis, CFTR, EMC

## Abstract

Osteoarthritis (OA) is a chronic degenerative joint disorder marked by progressive cartilage loss, synovial inflammation, and remodeling of the subchondral bone. Although roflumilast, a selective phosphodiesterase-4 inhibitor, has shown clinical benefits in chronic inflammatory diseases, its potential role in OA has not been defined. Here, we examined whether roflumilast can protect chondrocytes against apoptosis and extracellular matrix (ECM) degradation under inflammatory stress. In IL-1β–stimulated primary chondrocytes, roflumilast pretreatment (20 µM) suppressed the expression of matrix-degrading enzymes MMP13 and ADAMTS5, while restoring collagen II and aggrecan, thereby maintaining ECM integrity. Transcriptomic profiling identified a strong upregulation of cystic fibrosis transmembrane conductance regulator (CFTR) alongside the suppression of apoptosis-related genes. Mechanistically, roflumilast activated the Akt/Bcl-2 signaling cascade, and either pharmacological blockade or siRNA silencing of CFTR abolished these protective effects, indicating a CFTR-dependent pathway. Consistently, human OA cartilage displayed a marked reduction in CFTR expression, underscoring the clinical relevance of this mechanism. In vivo, roflumilast treatment in the destabilization of the medial meniscus mouse model alleviated cartilage erosion, improved subchondral bone architecture, and reduced synovial inflammation. Behavioral and imaging assessments further confirmed enhanced locomotor performance and mitigated joint degeneration. Collectively, our findings identify CFTR as a crucial mediator of roflumilast-induced chondroprotection and highlight roflumilast as a promising candidate for disease modification in OA.

**Supplementary Information:**

The online version contains supplementary material available at https://doi.org/10.1186/s13075-026-03872-0.

## Introduction

Osteoarthritis (OA) affects nearly 7% of the global population and is among the most common forms of arthritis [1]. It is characterized by articular cartilage erosion, synovial inflammation, and subchondral bone remodeling [2]. Current pharmacological options for early-stage OA, including nonsteroidal anti-inflammatory drugs (NSAIDs), diacerein, and glucosamine, offer only symptomatic relief without altering disease progression [3–5]. In advanced cases, joint replacement remains the definitive treatment [6]. OA pathogenesis reflects a complex interplay of mechanical stress, inflammation, and cellular dysfunction. Among these factors, interleukin-1β (IL-1β) plays a central role in disrupting chondrocyte homeostasis [7]. IL-1β promotes catabolism by inducing matrix metalloproteinase 13 (MMP13) and a disintegrin and metalloproteinase with thrombospondin motifs 5 (ADAMTS5), while simultaneously suppressing extracellular matrix (ECM) proteins such as collagen II and aggrecan. It also accelerates chondrocyte apoptosis, which correlates closely with the extent of cartilage destruction.

Apoptosis is a tightly regulated process essential for tissue homeostasis; however, excessive apoptosis drives tissue degeneration in several disorders, including OA [8]. The PI3K/Akt pathway is a key regulator of chondrocyte viability, acting through downstream targets such as Bcl-2 and caspase-3 [9–11]. Accordingly, targeting chondrocyte apoptosis under inflammatory conditions has emerged as a potential therapeutic approach.

Roflumilast, a selective phosphodiesterase 4 (PDE4) inhibitor approved for chronic obstructive pulmonary disease and psoriasis, elevates intracellular cAMP and modulates immune responses and cell survival. Previous studies have shown that roflumilast exerts anti-apoptotic effects in cardiomyocytes, granulosa cells, and neurons [12–16]. Whether similar protective effects occur in chondrocytes during OA progression remains unknown.

In this study, we investigated the role of roflumilast in inhibiting chondrocyte apoptosis and ECM degradation under IL-1β-induced inflammatory conditions. We further explored the involvement of cystic fibrosis transmembrane conductance regulator (CFTR) in mediating the anti-apoptotic effects of roflumilast. Our findings reveal that roflumilast protects chondrocytes and ameliorates OA progression both in vitro and in vivo by activating CFTR and the downstream Akt/Bcl-2 signaling pathway.

## Materials and methods

### Human cartilage sample collection

Six pairs of human cartilage samples were collected from patients with clinically diagnosed OA who underwent total knee replacement surgery (age range: 65–74 years, Kellgren–Lawrence grade IV). Patients with severe systemic diseases or major comorbidities were excluded. Sample collection was authorized by the Ethical Committee of the Nanjing Drum Tower Hospital, the Affiliated Hospital of Nanjing University Medical School (Approval number: 2022-176-01). All participants provided informed consent, and the study conformed to ethical guidelines.

### Animal model of destabilization of the medial meniscus (DMM)

Twelve-week-old male C57BL/6 mice weighing approximately 20–25 g and 3-day-old C57BL/6 mice were obtained from the Model Animal Research Center of Nanjing University (Jiangsu, China).

Mice were categorized into four groups (6 per group, 24 in total): [1] sham surgery (sham) [2], sham with roflumilast treatment (20 µM in DMSO-PBS, intra-articular injection every other day for 8 weeks) [3], DMM, and [4] DMM with roflumilast treatment (20 µM in DMSO-PBS, intra-articular injection every other day for 8 weeks). DMM surgery was performed according to established protocols [17], with the medial meniscal-tibial ligament sectioned to destabilize the medial meniscus in the right knee joint. A sham surgery was performed without ligament sectioning. The mice were sacrificed eight weeks postoperatively.

### Chondrocyte isolation and culture

Primary mouse chondrocytes were isolated from tibial plateaus and femoral heads of 3-day-old C57BL/6 mice and cultured according to established protocols [18]. Briefly, tissues were digested with collagenase (Gibco, Carlsbad, CA, USA) for 4 h at 37 °C. The isolated chondrocytes were then seeded at a density of 1 × 10^5 cells/well in DMEM/F12 medium (Gibco) supplemented with 10% FBS and 1% penicillin-streptomycin. Cells were maintained at 37 °C in a humidified incubator with 5% CO2. For experimental treatments, murine chondrocytes were exposed to 10 ng/mL IL-1β (#401-ML, R&D Systems), roflumilast (#S2131, Selleck), or 20 µM CFTR-inh172 (#S7139, Selleck). In the simultaneous treatment experiments, IL-1β and roflumilast were added at the same time and maintained for 48 h. In the delayed roflumilast treatment experiments, chondrocytes were first stimulated with 10 ng/mL IL-1β for 24 h, followed by the addition of roflumilast for another 48 h. CFTR-inh172 was used at a final concentration of 20 µM where indicated. CFTR-inh172 was used at a final concentration of 20 µM where indicated.

### Cell viability assay

Mouse primary chondrocytes were seeded into 96-well plates at a density of 5 × 10^3 cells/well. After 24 h of initial culture, cells were treated with fresh medium or varying concentrations of roflumilast for 48 h. Cell viability was assessed using the CCK-8 kit (#CK04, Dojindo) according to the manufacturer’s instructions. Briefly, 10 µL of CCK-8 solution was added to each well and incubated for 4 h at 37 °C. The absorbance was measured at 450 nm using a microplate reader.

### siRNA transfection

siRNA targeting the Cftr gene (sequence: 5‘-UUCCUGGACAGCUCAACUUUATT-3’) was synthesized by Nanjing Zebrafish Biotech, China. Chondrocytes were seeded at a density of 1 × 10^5 cells/well in a 6-well plate and allowed to reach 70% confluence. Transfection was performed using Lipo3000 (Thermo Fisher Scientific) with 50 nM siRNA-Cftr or a negative control siRNA according to the manufacturer’s protocol. Cells were incubated with the transfection mixture for 6 h, followed by replacement with fresh complete medium. Subsequent experiments were performed 12 h after transfection.

### Western blot

Western blot analysis was performed as previously described [19]. Briefly, total protein was extracted from chondrocytes using RIPA lysis buffer (Thermo Fisher Scientific) supplemented with protease and phosphatase inhibitors]. Protein concentrations were determined using the BCA protein assay kit (Thermo Fisher Scientific)]. 20–30 µg of total protein per sample was separated by 10% SDS-PAGE and then transferred onto PVDF membranes. Membranes were blocked with 5% non-fat milk or BSA in TBST for 1 h at room temperature and then incubated overnight at 4 °C with primary antibodies: Col2 (#43306S, Cell Signaling Technology, 1:1000), Aggrecan (#A12045, Abclonal, 1:1000), MMP13 (#GB11247-1-100, Servicebio, 1: 1000), Adamts5 (#A2836, Abclonal, 1: 1000), Bax (#60267-1-Ig, Proteintech, 1: 1000), Bcl2 (#15071S, Cell Signaling Technology, 1: 1000), Akt (#9272S, Cell Signaling Technology, 1: 1000), p-AKT (#9271S, Cell Signaling Technology, 1: 1000), Caspase 3 (#9662S, Cell Signaling Technology, 1: 1000), Cftr (#20738-1-AP, Proteintech, 1: 1000), and Gapdh (#60004-1-Ig, Proteintech, 1: 1000). After washing, membranes were incubated with HRP-conjugated secondary antibodies (#BL003A or #BL001A, Biosharp, 1:5000]) for 1 h at room temperature. Protein bands were visualized using an enhanced chemiluminescence (ECL) detection system (Abbkine) and images were captured. Densitometric analysis was performed using ImageJ software.

### ELISA

Intracellular cAMP levels were measured in primary chondrocytes using a cAMP ELISA Kit (#KGE012B, R&D Systems). The results were analyzed using standard curves. The mean values from duplicate tests were used for statistical analysis.

### Quantitative real-time polymerase chain reaction (qPCR)

Total RNA was extracted from chondrocytes using the RNA-Quick Purification Kit (#RN001, ES Science, Shanghai, China) following the manufacturer’s protocol. RNA concentration and purity were determined using a NanoDrop spectrophotometer]. 1 µg of total RNA was reverse-transcribed into complementary DNA (cDNA) using the PrimeScript RT Reagent Kit (Vazyme) according to the manufacturer’s instructions. qPCR was performed using 20 µL reaction mixtures containing diluted cDNA, SYBR Green Master Mix (#Q411-02, Vazyme, Nanjing, China), and gene-specific primers on a LightCycler 480 System (Roche, Switzerland). The cycling conditions were: initial denaturation at 95 °C for 5 min, followed by 40 cycles of 95 °C for 15 s and 60 °C for 30 s. Relative gene expression was calculated using the 2**⁻**ΔΔCt method with Gapdh as an internal control. Primer sequences are listed in Supplementary Table 1.

### TdT-mediated dUTP nick-end labeling (TUNEL)

Chondrocyte apoptosis was assessed using the TUNEL assay. For in vitro studies, pretreated chondrocytes were fixed with 4% paraformaldehyde for 30 min and permeabilized with 0.1% Triton X-100]. For tissue sections, deparaffinized knee joint slides were used. Both chondrocytes and tissue sections were then incubated with the TUNEL reaction mixture (#KTA2010, Abbkine) for 1 h at 37 °C in the dark. Nuclei were counterstained with DAPI (#ab104139, Abcam). Images were obtained using a fluorescence microscope (Zeiss Inc., Heidelberg) and analyzed using ImageJ software.

### Flow cytometry

Flow cytometry was performed to detect apoptosis using a commercial Annexin V-FITC/PI Apoptosis Detection Kit (Abbkine]). Treated chondrocytes were collected by trypsinization, washed twice with cold PBS, and then resuspended in 100 µL of 1X binding buffer at a concentration of 1 × 10^6 cells/mL]. Subsequently, 5 µL of propidium iodide (PI) and 10 µL of FITC-conjugated Annexin V were added to the cell suspension and incubated in the dark for 15 min at room temperature. Apoptotic cells were analyzed using a BD Accuri C6 flow cytometer (BD, USA). Data were analyzed using FlowJo software.

### Intracellular Cl⁻ concentration measurements

Chondrocytes were incubated with 5 mM MQAE (#HY-D0090, MedChemExpress) for 30 min and then rinsed with PBS. Fluorescence, reflecting Cl⁻ concentration, was observed using a confocal fluorescence microscope (Zeiss Inc., Heidelberg). Finally, the results were analyzed using the ImageJ software.

### RNA sequencing

Targeted gene sequencing was conducted on the Illumina NovaSeq 6000 platform by Shanghai Personal Biotechnology Co., Ltd. After raw data processing, gene expression levels were calculated via HTSeq (v0.9.1) and normalized to FPKM. Differentially expressed genes (DEGs) were identified using DESeq (v1.39.0), with filtering criteria of |log₂FoldChange| > 1 and *P* < 0.05. Heatmaps and volcano plots were generated to visualize DEG expression patterns using R packages pheatmap (v1.0.12) and ggplot2, respectively. For heatmap construction, bidirectional hierarchical clustering of DEGs was performed: distances between genes and samples were calculated by the Euclidean method, and complete linkage was used for clustering, based on gene expression patterns across and within samples. Finally, Gene Ontology (GO) functional annotation and Kyoto Encyclopedia of Genes and Genomes (KEGG) pathway enrichment analysis of DEGs were performed using the clusterProfiler R package, with results filtered at *P* < 0.05 [20].

### Open field test and footprint experiment

Each mouse was placed in a 50 cm × 50 cm square arena, and its activity was monitored for 3 min to measure relative activity, active time, distance traveled, and mean speed. For the footprint experiment, the front and hind paws of the mice were dipped in different colored inks, and their walking trajectories were recorded on white paper as they traversed a 70 cm-long, 20 cm-wide track. The test was conducted in a quiet, dark environment with each mouse tested three times. The ink used was nontoxic.

### Micro-computed tomography (Micro-CT) analysis

Mice knee joints were analyzed. The joints were scanned at an 18.38 mm resolution, and 3D images were reconstructed using Scanco Medical software. Cancellous bone parameters, subchondral bone volume, number of bone fragments, and subchondral bone thickness were calculated using 3D reconstruction.

### Histological analysis

After micro-CT, knee joints were decalcified in 10% EDTA (#1340, Biofroxx), embedded in paraffin, sectioned into 5-µm thick slices, and stained with Toluidine Blue (#G2543, Solarbio) and H&E (#C0105M, Beyotime) to assess cartilage lesions and synovitis. Cartilage destruction was scored using the Osteoarthritis Research Society International (OARSI) grading system (0–6), and synovitis was scored on a 0–3 scale [21]. The mean scores were calculated.

### Immunofluorescence (IF) and immunohistochemical (IHC) staining

For cytological immunofluorescence, chondrocytes were seeded on coverslips at a density of 5 × 10^4 cells/well. After treatment, cells were fixed with 4% paraformaldehyde for 30 min, permeabilized with 0.1% Triton X-100 for 30 min, and blocked with 5% BSA in PBS for 1 h at room temperature. Slides were then incubated overnight at 4 °C with primary antibodies: Col2 (#BA0533, Boster, 1:200), Aggrecan (#A12045, Abclonal, 1:200), MMP13 (#ab39012, Abcam, 1:200), and Adamts5 (ab246975, Abcam, 1:200). After three washes with PBS, slides were incubated with FITC- or tetramethylrhodamine-conjugated secondary antibodies (Abcam, 1:200) for 1 h at room temperature in the dark. Nuclei were stained with DAPI. Images were captured using a fluorescence microscope (Zeiss, Inc., Heidelberg).

For histological immunofluorescence, deparaffinized knee joint slides were blocked with 5% BSA for 1 h and incubated overnight at 4 °C with primary antibodies against MMP13 (#ab39012, Abcam, 1:200) and Adamts5 (ab246975, Abcam, 1:200]). After three washes with PBS, slides were incubated with FITC-conjugated secondary antibodies (Abcam, 1:200) for 1 h at room temperature in the dark. Nuclei were stained with DAPI. The images were captured using a fluorescence microscope (Zeiss Inc., Heidelberg).

For immunohistochemistry, deparaffinized slides were treated with 3% H2O2 for 15 min to quench endogenous peroxidase activity, followed by antigen retrieval using pepsin (0.1%, 1 h). The slides were blocked with 5% BSA for 1 h and incubated overnight at 4 °C with primary antibodies against Col2 (#BA0533, Boster, 1:200) and Aggrecan (#13880-1-AP, Proteintech, 1:200). After washing, slides were incubated with HRP-coupled secondary antibodies (biosharp, 1:200) for 1 h. Color development was performed using an ultrasensitive DAB kit (#1 205 250, Typing) for 5 min. The slides were counterstained with hematoxylin, dehydrated, and sealed with resin. IHC images were acquired using a fluorescence microscope (Zeiss, Inc., Heidelberg).

### Statistical analysis

Statistical analyses were conducted using GraphPad Prism software (version 9.0). Quantitative data represent the results of at least three independent experiments. Comparisons between two groups were made using a two-tailed Student’s t-test (paired or unpaired as appropriate). For comparisons among multiple groups, one-way ANOVA followed by Tukey’s post hoc test was used to identify significant differences between specific groups. A p-value < 0.05 was considered statistically significant. Data are expressed as means ± standard deviation.

## Results

### Roflumilast attenuates ECM degradation and apoptosis in IL-1β-stimulated chondrocytes

**Fig. 1:**
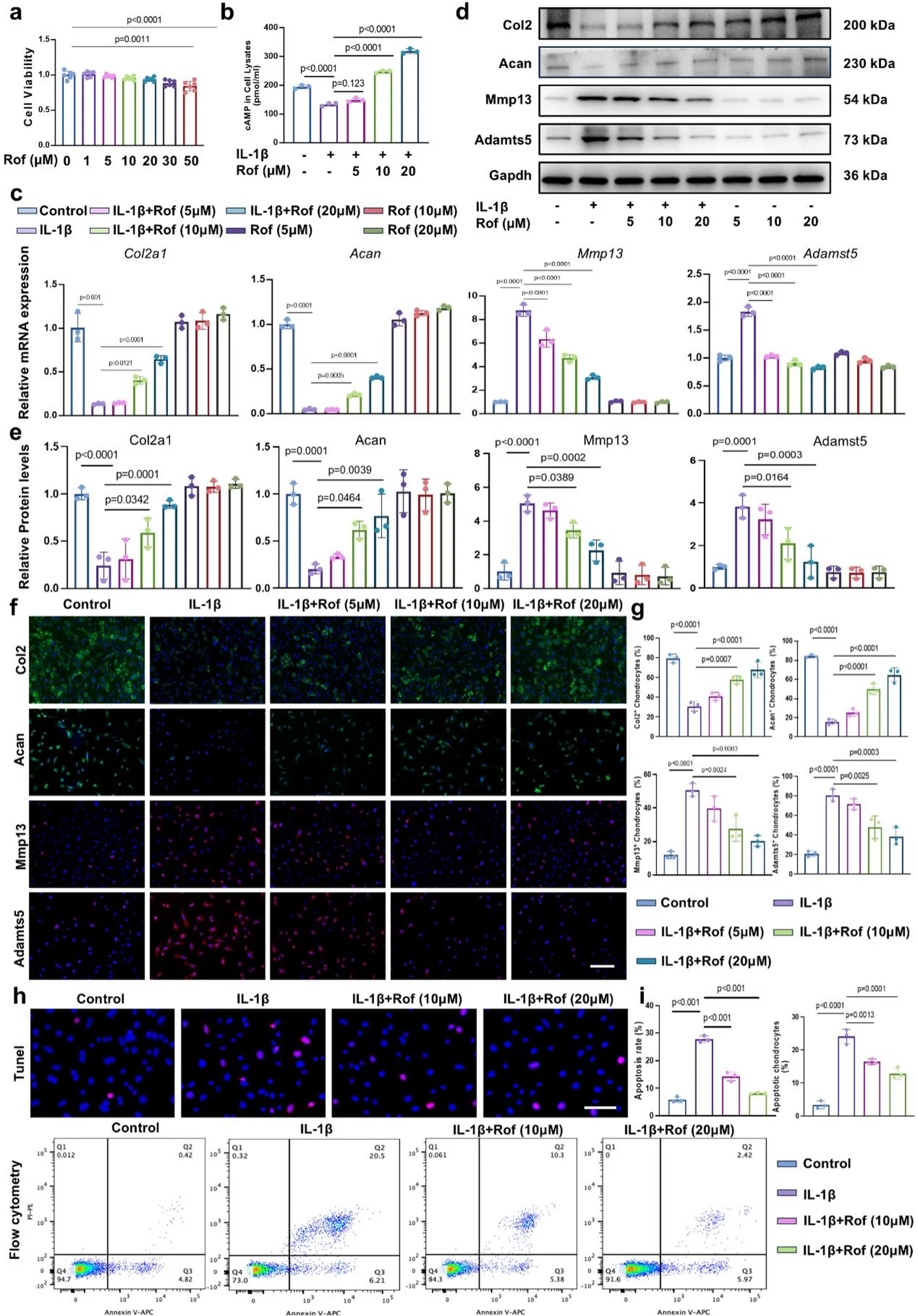
Roflumilast attenuates ECM degradation and apoptosis in IL-1β-stimulated chondrocytes. **a** Cell viability of primary murine chondrocytes treated with increasing concentrations of roflumilast (0, 1, 5, 10, 20, 30, and 50 μM) for 48 h. Data are expressed as means ± SD. Statistical analysis was performed using one-way ANOVA followed by Tukey’s post hoc test for comparisons among multiple groups. **b** Intracellular cAMP levels in primary murine chondrocytes treated with roflumilast. Data are expressed as means ± SD. Statistical analysis was performed using one-way ANOVA followed by Tukey’s post hoc test for comparisons among multiple groups. **c** mRNA expression of Adamts5, Mmp13, Col2, and Acan in IL-1β-stimulated chondrocytes with or without roflumilast treatment, determined by qPCR. Data are expressed as means ± SD. Statistical analysis was performed using one-way ANOVA followed by Tukey’s post hoc test for comparisons among multiple groups. **d**-**e** Protein expression of Adamts5, Mmp13, Col2, and Acan in IL-1β-stimulated chondrocytes with or without roflumilast treatment, determined by Western blot. Data are expressed as means ± SD. Statistical analysis was performed using one-way ANOVA followed by Tukey’s post hoc test for comparisons among multiple groups. **f**-**g** Immunofluorescence staining for Col2, Acan, Mmp13, and Adamts5 in IL-1β-stimulated chondrocytes with or without roflumilast treatment. Data are expressed as means ± SD. Statistical analysis was performed using one-way ANOVA followed by Tukey’s post hoc test for comparisons among multiple groups. **h**-**i** TUNEL staining and flow cytometry for apoptosis in IL-1β-stimulated chondrocytes with or without roflumilast treatment. Data are expressed as means ± SD. Statistical analysis was performed using one-way ANOVA followed by Tukey’s post hoc test for comparisons among multiple groups. *P < 0.05 was considered statistically significant

To evaluate the chondroprotective effects of roflumilast, we first assessed its impact on viability and ECM homeostasis in primary murine chondrocytes. Cells were treated with increasing concentrations of roflumilast (0, 1, 5, 10, 20, 30, and 50 µM) for 48 h, and viability remained unaffected at doses up to 20 µM (Fig. 1a). Based on these results, 5, 10, and 20 µM were selected for subsequent experiments. Roflumilast was dissolved in DMSO and diluted to the indicated concentrations before use. A vehicle control containing 0.2% DMSO, the highest final DMSO concentration in the roflumilast-treated groups, was included. The CCK-8 assay confirmed that 0.2% DMSO did not significantly affect mouse chondrocyte viability (Supplementary Fig. 1). As expected for a PDE4 inhibitor, roflumilast significantly elevated intracellular cAMP levels in a dose-dependent manner (Fig. 1b).

Further analyses were conducted to investigate the effects of roflumilast on ECM degradation markers. Quantitative real-time PCR (qPCR) demonstrated that IL-1β stimulation significantly induced the mRNA expression of Adamts5 and Mmp13 (*p* < 0.001 vs. control), while significantly suppressing the mRNA expression of Col2 and Acan (*p* < 0.001 vs. control). Roflumilast treatment (5, 10, 20 µM) dose-dependently reversed these catabolic changes, significantly reducing Adamts5 and Mmp13 mRNA levels (*p* < 0.001 vs. IL-1β group). Meanwhile, roflumilast treatment (10, 20 µM) increased Col2 (*p* = 0.0121, *p* < 0.001 vs. IL-1β group) and Acan (*p* < 0.005, *p* < 0.001 vs. IL-1β group) mRNA levels (Fig. 1c). The results of western blot confirmed these findings at the protein level. IL-1β treatment significantly increased Adamts5 and Mmp13 protein expression (*p* < 0.0001 vs. control) and decreased Col2 and Acan protein expression (*p* < 0.0001 vs. control). Roflumilast treatment(10, 20 µM) significantly attenuated the IL-1β-induced upregulation of Adamts5 protein (*p* = 0.0164, *p* = 0.0003 vs. IL-1β group) and Mmp13 protein (*p* = 0.0389, *p* = 0.0002 vs. IL-1β group), and restored the expression of Col2 protein (*p* = 0.0342, *p* = 0.0001 vs. IL-1β group) and Acan protein (*p* = 0.0464, *p* = 0.0039 vs. IL-1β group) in a dose-dependent manner (Fig. 1d–e). Immunofluorescence staining further supported the preservation of ECM integrity. Chondrocytes treated with IL-1β showed reduced fluorescence intensity for Col2 and Acan, and increased intensity for Mmp13 and Adamts5. Roflumilast treatment significantly enhanced Col2 fluorescence intensity (*p* = 0.0007, *p* < 0.0001 vs. IL-1β group) and Acan fluorescence intensity (*p* < 0.0001, *p* < 0.0001 vs. IL-1β group), and reduced Mmp13 intensity (*p* = 0.0024, *p* = 0.0003 vs. IL-1β group) and Adamts5 intensity (*p* = 0.0003, *p* = 0.0025 vs. IL-1β group), indicating a restoration of ECM components (Fig. 1f–g).

To further determine whether roflumilast exerts protective effects after inflammatory injury has been established, chondrocytes were first stimulated with IL-1β for 24 h and then treated with roflumilast. Roflumilast increased COL2 expression and reduced MMP13 expression in this post treatment model (Supplementary Fig. 2), suggesting that roflumilast can attenuate ECM degradation even after IL-1β induced inflammatory injury has occurred.

To assess chondrocyte apoptosis, TUNEL staining and flow cytometry were performed. IL-1β stimulation significantly increased the percentage of TUNEL-positive chondrocytes (*p* < 0.001 vs. control) and the apoptotic cell population as determined by flow cytometry (*p* < 0.001 vs. control). Roflumilast treatment significantly inhibited IL-1β–induced chondrocyte apoptosis, leading to a significant reduction in TUNEL-positive cells (*p* = 0.0013, *p* = 0.0001 vs. IL-1β group) and apoptotic cell percentage (*p* < 0.001, *p* < 0.001 vs. IL-1β group) (Fig. 1h–i).

### Roflumilast improves behavioral performance in OA mice

**Fig. 2:**
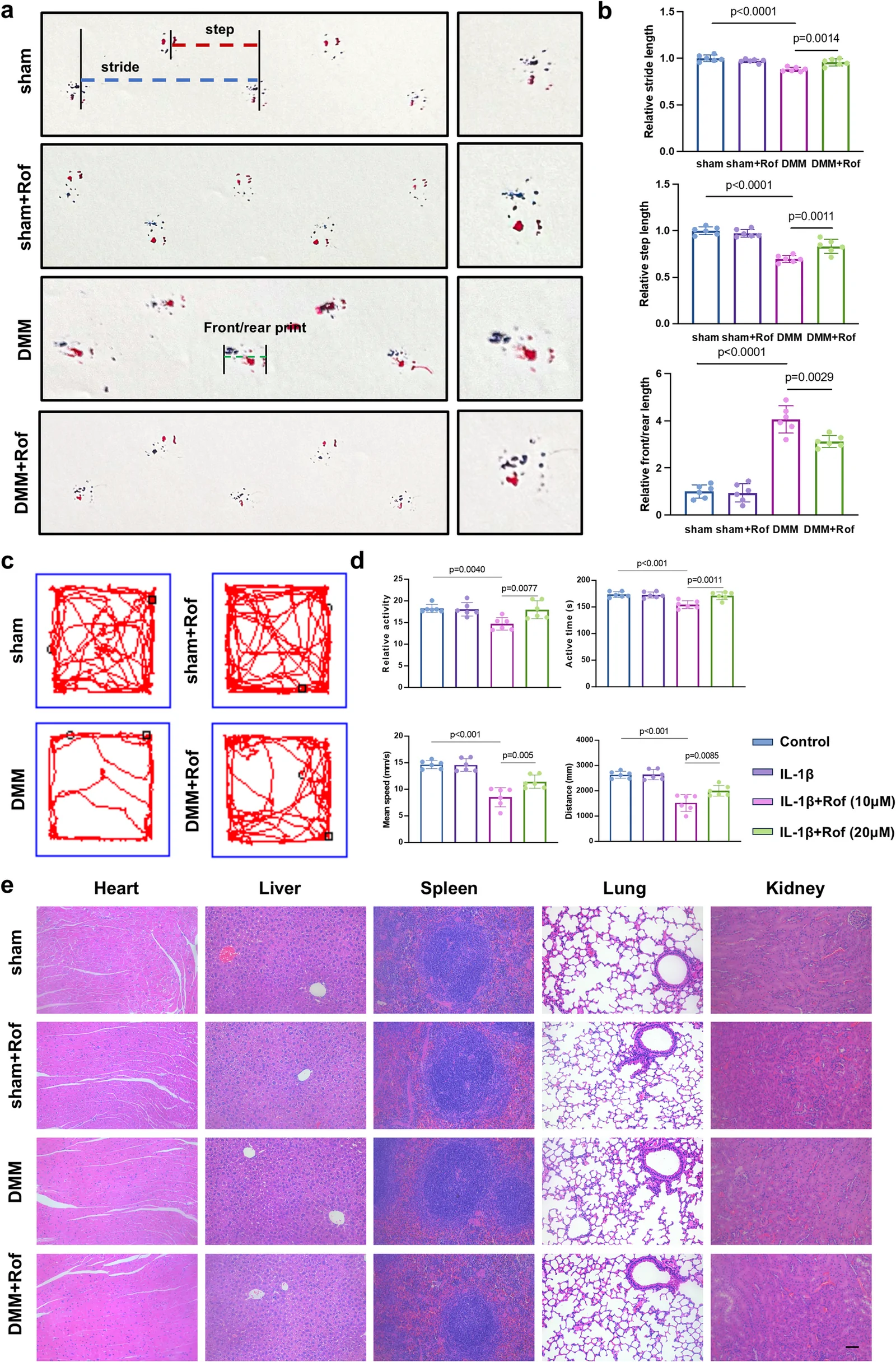
Roflumilast improved behavioral performance in OA mice. **a**-**b** Footprint analysis showing stride length, step length, and discrepancy between front and rear paw prints in sham, DMM, and DMM with roflumilast treatment groups. Data are expressed as means ± SD (n=6 per group). Statistical analysis was performed using one-way ANOVA followed by Tukey’s post hoc test for comparisons among multiple groups. **c**-**d** Open field test results measuring relative activity, active time, distance traveled, and mean speed in sham, DMM, and DMM with roflumilast treatment groups. Data are expressed as means ± SD (n=6 per group). Statistical analysis was performed using one-way ANOVA followed by Tukey’s post hoc test for comparisons among multiple groups. (e) Representative H&E staining of various tissues from sham, DMM, and DMM with roflumilast treatment groups, indicating the absence of overt toxicity. *P < 0.05 was considered statistically significant

To explore the potential role of roflumilast in vivo, a DMM model of OA was utilized. The experimental mice were divided into four groups: sham, roflumilast-treated sham, DMM, and roflumilast-treated DMM. The effect of roflumilast on OA symptoms was assessed using footprint experiments to evaluate pain and gait abnormalities in DMM mice [22]. The results of footprint analysis indicated that roflumilast treatment significantly improved stride length (*p* = 0.0014 vs. DMM group) and step length (*p* = 0.0011 vs. DMM group), while reducing the discrepancy between the front and rear paw prints (*p* = 0.0029 vs. DMM group) (Fig. 2a, b). Furthermore, we assessed voluntary activities over a 3-min period. Compared with the sham group, the DMM group exhibited notable reductions in relative activity, active time, distance traveled, and mean speed (*p* = 0.004, *p* < 0.001, *p* < 0.001, *p* < 0.001 vs. sham group). However, these parameters significantly improved following roflumilast treatment (*p* = 0.0077 *p* = 0.0011, *p* = 0.005 *p* < 0.0085 vs. DMM group) (Fig. 2c-d). Overall, roflumilast treatment improved locomotor performance and enhanced spontaneous movement in DMM mice, thereby significantly alleviating the activity limitations associated with OA. Whether this functional improvement is associated with reduced pain perception requires further investigation. Moreover, H&E staining of several tissues revealed no significant structural or cellular differences between the groups (Fig. 2e), indicating the absence of overt toxicity associated with roflumilast treatment.

### Roflumilast ameliorates cartilage erosion and alleviates OA progression

To comprehensively evaluate the therapeutic impact of roflumilast on OA, we performed an in-depth analysis of bone remodeling within the knee joints of mice. Three-dimensional reconstruction of knee joints using micro-CT revealed a significant increase in osteophyte formation and subchondral bone sclerosis in DMM-treated mice, particularly in the medial aspect (*p* < 0.0001 vs. sham group).

**Fig. 3:**
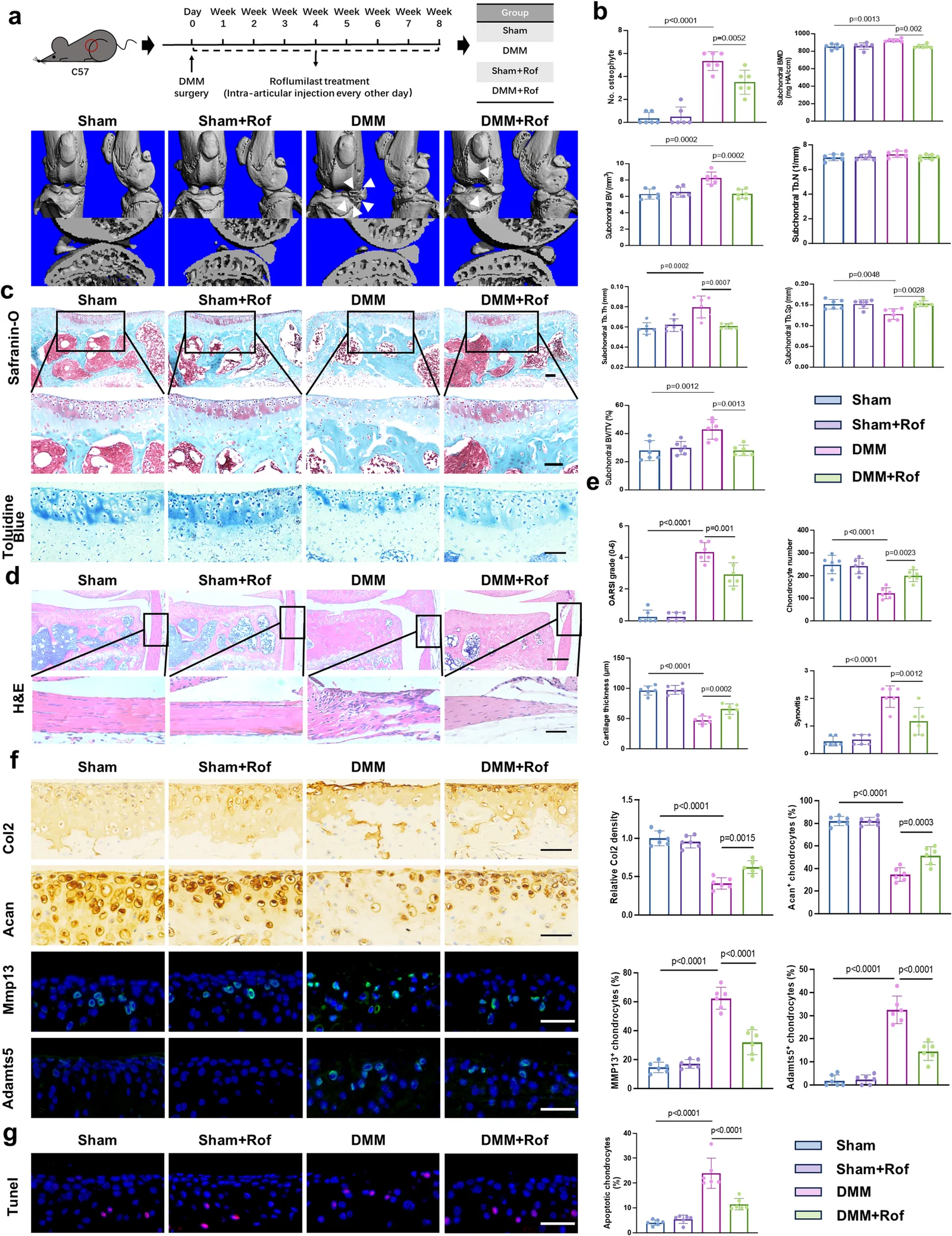
Roflumilast ameliorates cartilage erosion and alleviates OA progression. **a**-**b** Micro-CT analysis showing osteophyte formation, subchondral bone sclerosis, osteophyte numbers, subchondral bone volume, bone mineral density, trabecular thickness (Tb.Th), and trabecular separation (Tb.Sp) in sham, DMM, and DMM with roflumilast treatment groups. Data are expressed as means ± SD (n=6 per group). Statistical analysis was performed using one-way ANOVA followed by Tukey’s post hoc test for comparisons among multiple groups. **c**-**e** Toluidine blue staining, OARSI scores, chondrocyte number, cartilage thickness, and synovitis scores in sham, DMM, and DMM with roflumilast treatment groups. Data are expressed as means ± SD (n=6 per group). Statistical analysis was performed using one-way ANOVA followed by Tukey’s post hoc test for comparisons among multiple groups. **f** Immunofluorescence staining for Col2/Acan and Mmp13/Adamts5 expression in DMM and DMM with roflumilast treatment groups. Data are expressed as means ± SD (n=6 per group). Statistical analysis was performed using one-way ANOVA followed by Tukey’s post hoc test for comparisons among multiple groups. **g** TUNEL staining for chondrocyte apoptosis in DMM and DMM with roflumilast treatment groups. Data are expressed as means ± SD (n=6 per group). Statistical analysis was performed using one-way ANOVA followed by Tukey’s post hoc test for comparisons among multiple groups. *P < 0.05 was considered statistically significant

To comprehensively evaluate the therapeutic impact of roflumilast on OA, we performed an in-depth analysis of bone remodeling within the knee joints of mice. Three-dimensional reconstruction of knee joints using micro-CT revealed a significant increase in osteophyte formation and subchondral bone sclerosis in DMM-treated mice, particularly in the medial aspect. In addition, both the tibia and femur exhibited irregular bone surfaces with elevated osteophyte numbers, subchondral bone volume, and bone mineral density (*p* < 0.0001, *p* = 0.002, *p* = 0.0013 vs. sham group) (Fig. 3a–b). Treatment with roflumilast significantly ameliorated these pathological changes, leading to a significant reduction in osteophyte numbers, subchondral bone volume, and bone mineral density (*p* = 0.0052, *p* = 0.0013, *p* = 0.002 vs. DMM group). Specifically, DMM-induced mice displayed enhanced trabecular thickness (Tb.Th) and reduced trabecular separation (Tb.Sp) compared to the sham group (*p* = 0.0002, *p* = 0.0048 vs. sham group). Roflumilast treatment significantly reversed these changes, increasing Tb.Th and decreasing Tb.Sp (*p* = 0.0007, *p* = 0.0028 vs. DMM group) (Fig. 3b). These results supported that subchondral bone abnormalities caused by OA were significantly improved after administering roflumilast.

Safranin O/Fast Green staining and Toluidine blue staining showed less cartilage erosion in the roflumilast-treated DMM group than in the DMM group. Consistently, roflumilast-treated DMM mice exhibited lower OARSI scores, a higher number of chondrocytes, and increased cartilage thickness compared with DMM mice (*p* = 0.001, *p* = 0.0023, *p* = 0.0002 vs. DMM group) with decreased cartilage erosion (Fig. 3c–e). Synovitis scores were also significantly decreased in the roflumilast-treated DMM group (*p* = 0.0012 vs. DMM group) (Fig. 3d–e). Immunofluorescence suggested restoration of cartilage metabolic balance, with significantly higher expression of Col2 and Acan (*p* = 0.0015, *p* = 0.0003 vs. DMM group) and lower expression of Mmp13 and Adamts5 (*p* < 0.0001, *p* < 0.0001 vs. DMM group) in the roflumilast-treated DMM group (Fig. 3f). TUNEL staining further showed significantly reduced chondrocyte apoptosis in roflumilast-treated OA mice (*p* < 0.0001 vs. DMM group) (Fig. 3g). These results collectively suggest that roflumilast protected against OA-associated cartilage degeneration and synovial inflammation.

### Transcriptomic analysis reveals CFTR as a key downstream effector of roflumilast

**Fig. 4:**
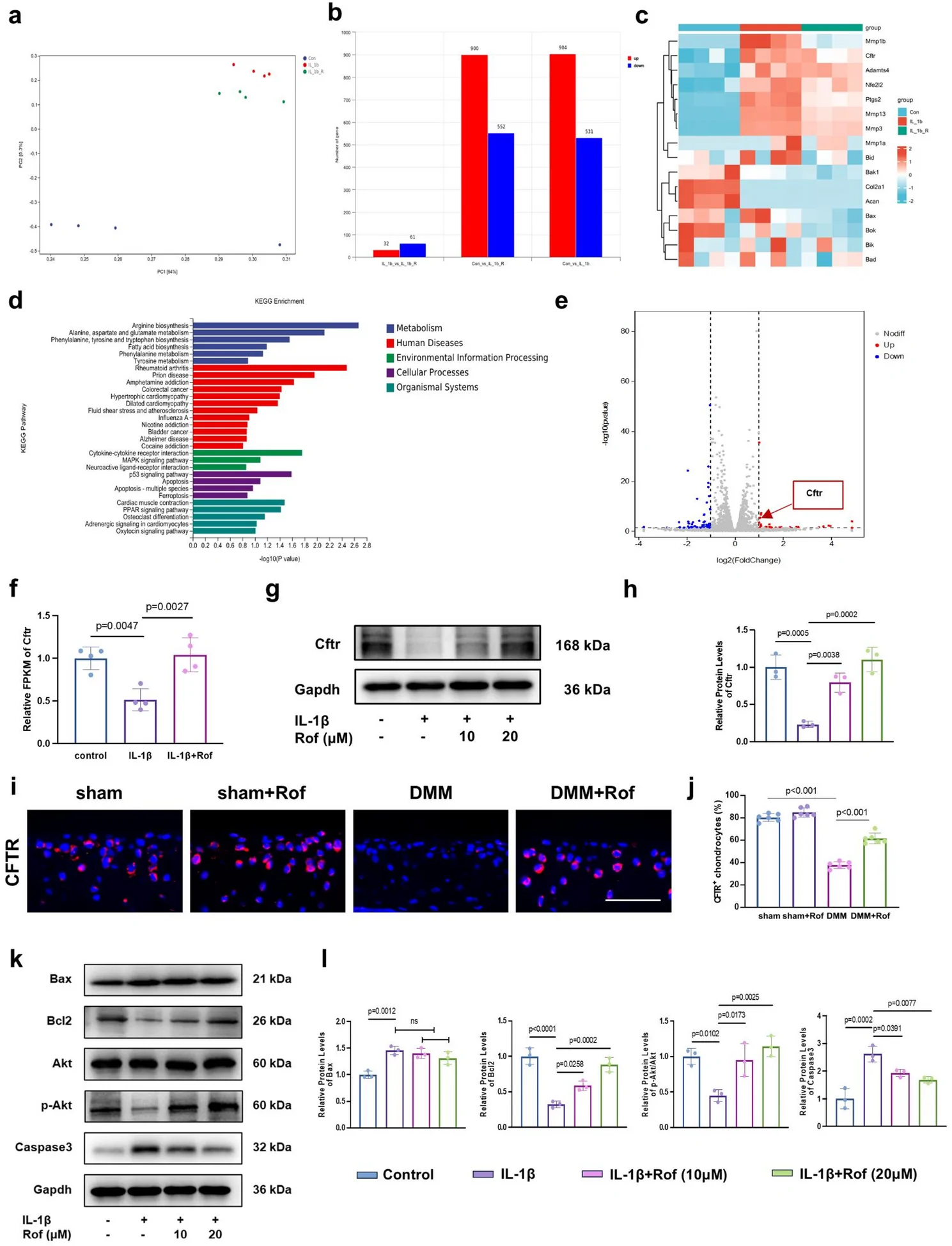
Transcriptomic analysis reveals CFTR as a key downstream effector of roflumilast. **a**-**b** Volcano plots illustrating differential gene expression in control, IL-1β-induced, and IL-1β-induced Roflumilast-treated chondrocytes. Differentially expressed genes were selected based on |log2FoldChange| > 1 and P < 0.05. **c**-**d** Heatmap and KEGG pathway analysis showing significant enrichment in pathways related to apoptosis, ferroptosis, p53, MAPK, and PPAR signaling. **e**-**f** Data indicating CFTR upregulation following roflumilast treatment. **g**-**j** Immunoblotting showing increased CFTR protein expression both in vitro and in vivo. Data are expressed as means ± SD. Statistical analysis was performed using one-way ANOVA followed by Tukey’s post hoc test for comparisons among multiple groups. **k**-**l** Western blot analysis showing Bcl-2, p-Akt/AKT, and Caspase 3 expression in IL-1β-stimulated chondrocytes with or without roflumilast treatment. Data are expressed as means ± SD. Statistical analysis was performed using one-way ANOVA followed by Tukey’s post hoc test for comparisons among multiple groups. *P < 0.05 was considered statistically significant

To validate the potential mechanisms driving the chondroprotective effects of roflumilast, we conducted whole transcriptome RNA sequencing on the control, IL-1β-induced, and IL-1β-induced Roflumilast-treated chondrocytes. We performed differential expression analysis of gene expression using DESeq, calculating the fold change and significance P-value for each gene. Differentially expressed genes were selected based on the following criteria: |log2FoldChange| > 1 and significance P-value < 0.05(Fig. 4a-b). The Heatmap and KEGG pathway analysis (IL-1β + Rof vs. IL-1β) revealed significant enrichment in pathways related to apoptosis, ferroptosis, p53, MAPK, and PPAR signaling (Fig. 4c-d). Among the differentially expressed DEGs, the cystic fibrosis transmembrane conductance regulator (CFTR) was one of the most upregulated genes following roflumilast treatment (Fig. 4e–f). Immunoblotting indicated increased CFTR protein expression both in *vitro* and in *vivo* (Fig. 4g–i). Based on existing literature and our working hypothesis, we postulated that CFTR enhances OA outcomes by upregulating the Akt/Bcl2-mediated anti-apoptotic pathway. To verify this hypothesis, we assessed the effect of roflumilast on chondrocyte apoptosis. Western blot analysis indicated that Roflumilast, in a dose-dependent manner (10, 20 µM), increased p-Akt/ Akt and Bcl-2 expression (*p* = 0.0173, *p* = 0.0025 vs. IL-1β group for p-Akt/Akt; *p* = 0.0258, *p* = 0.0002 vs. IL-1β group for Bcl-2) while reducing the expression of Bax and Caspase 3 (*p* = 0.0391, *p* = 0.0077 vs. IL-1β group for Caspase 3) (Fig. 4k–l). IL-1β treatment significantly elevated the levels of apoptotic proteins, including Caspase 3(*p* = 0.0002 vs. control), while concurrently reducing the expression of p-Akt/AKT (*p* = 0.0102 vs. control) and Bcl-2(*p* < 0.0001 vs. control). Roflumilast treatment effectively restored these levels.

### CFTR expression is decreased in human OA cartilage

**Fig. 5:**
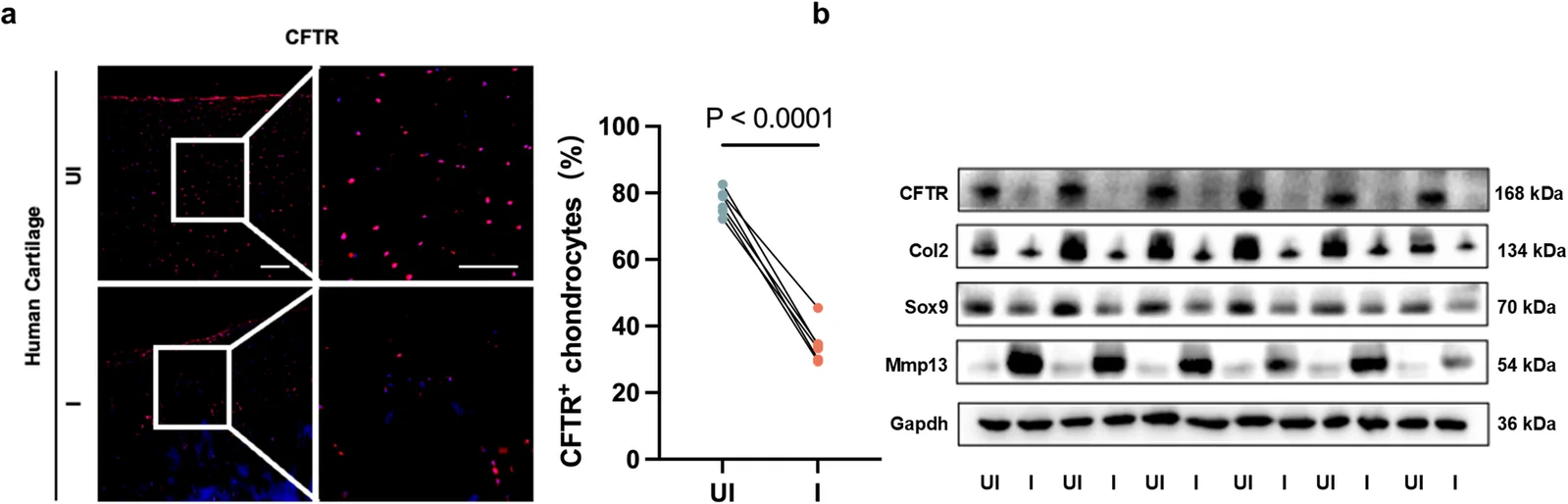
CFTR expression is decreased in human OA cartilage. **a** Immunofluorescence staining revealing CFTR expression in injured regions compared with uninjured regions from the same human OA patients (n=6). Statistical analysis was performed using a two-tailed Student’s t-test (paired). **b** Western blot analysis confirming decreased CFTR protein levels in injured areas of paired human OA cartilage tissues. Data are expressed as means ± SD (n=6). Statistical analysis was performed using a two-tailed Student’s t-test (paired)

To evaluate the clinical relevance of CFTR in OA, we examined its expression in human articular cartilage samples using immunofluorescence staining. Compared with matched uninjured regions from the same OA patients, injured cartilage regions showed a reduced proportion of CFTR-positive chondrocytes (*n* = 6) (Fig. 5a). This reduction appeared to be more prominent in the middle zone of the cartilage. Western blot analysis of paired cartilage tissues further confirmed the decreased CFTR protein levels in injured areas (Fig. 5b). These findings demonstrated that CFTR downregulation is closely associated with cartilage degeneration in human OA, supporting the translational relevance of our mechanistic observations.

### Roflumilast inhibits chondrocyte apoptosis via CFTR/Akt/Bcl2 signaling

**Fig. 6:**
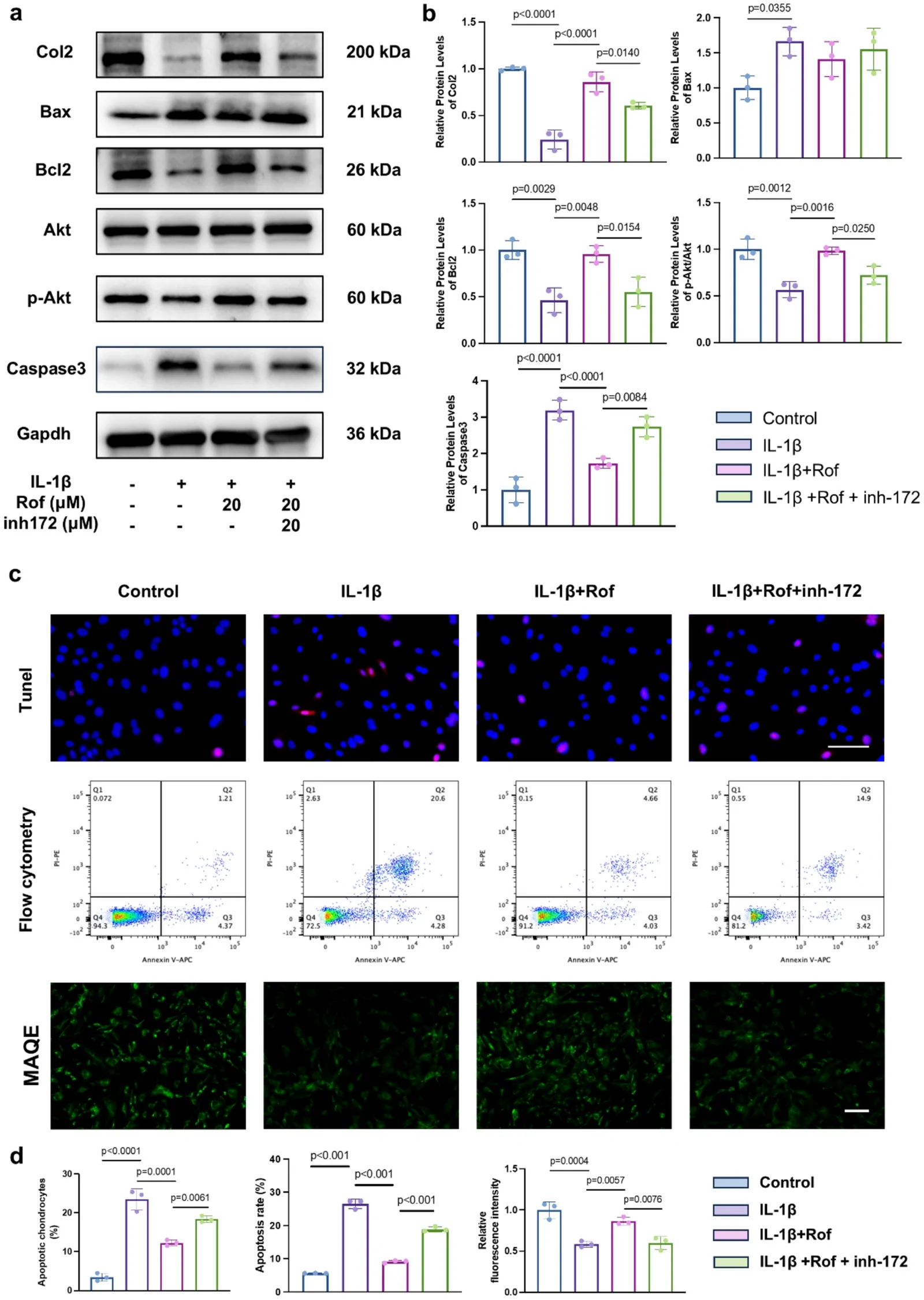
Inhibition of CFTR disables roflumilast resistance to chondrocyte apoptosis. **a**-**b** Western blot analysis showing p-Akt, Caspase 3, and Bcl-2 expression in IL-1β-stimulated chondrocytes treated with roflumilast, and with or without CFTRinh-172. Data are expressed as means ± SD. Statistical analysis was performed using one-way ANOVA followed by Tukey’s post hoc test for comparisons among multiple groups. **c**-**d** TUNEL staining and flow cytometry assays for apoptosis in IL-1β-stimulated chondrocytes treated with roflumilast, and with or without CFTRinh-172. Data are expressed as means ± SD. Statistical analysis was performed using one-way ANOVA followed by Tukey’s post hoc test for comparisons among multiple groups. **c**-**d** Intracellular chloride concentrations measured by MQAE in IL-1β-stimulated chondrocytes treated with roflumilast, and with or without CFTRinh-172. Data are expressed as means ± SD. Statistical analysis was performed using one-way ANOVA followed by Tukey’s post hoc test for comparisons among multiple groups. *P < 0.05 was considered statistically significant

**Fig. 7:**
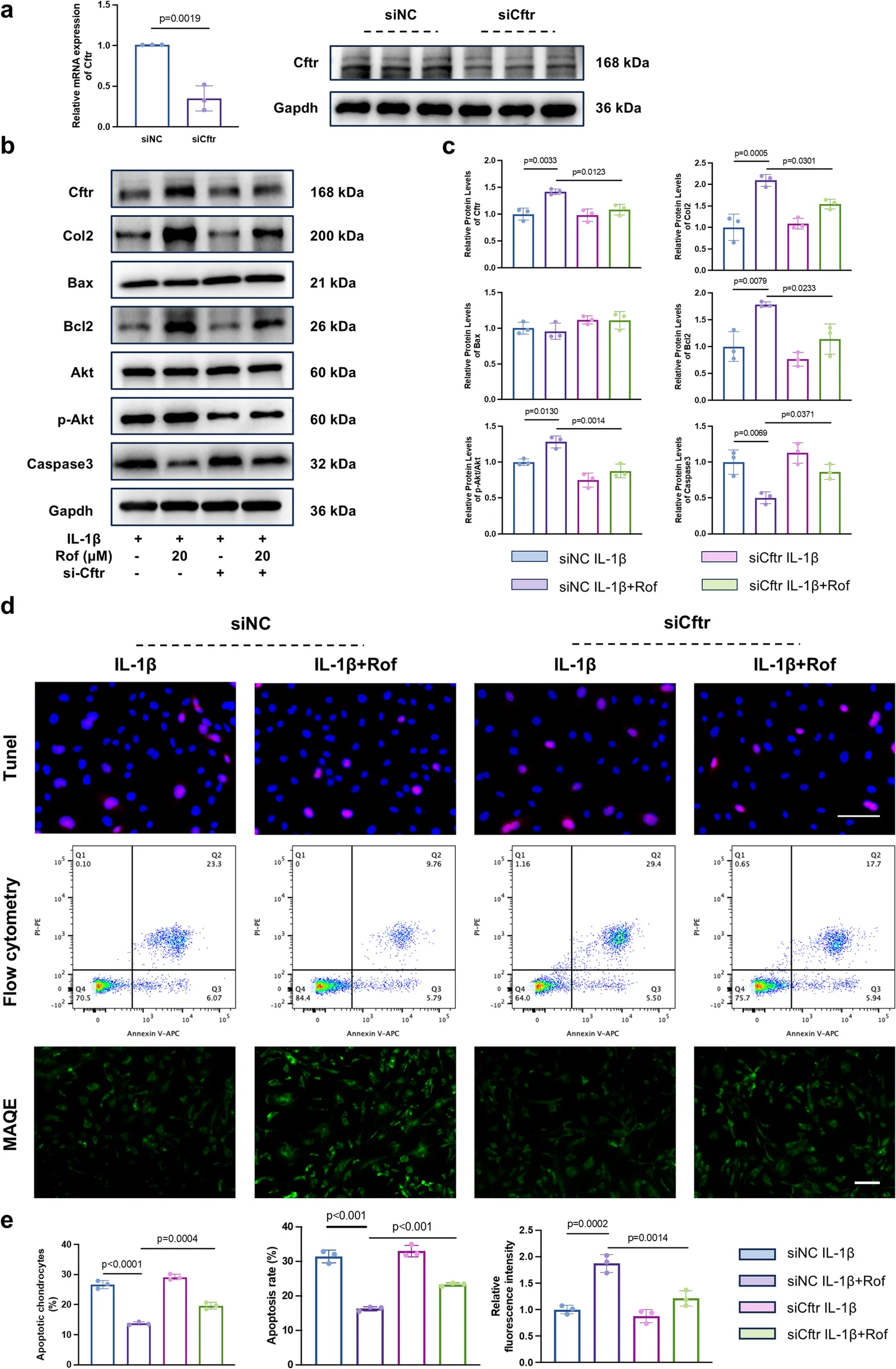
CFTR mediates the attenuation of chondrocyte apoptosis by Roflumilast. **a** QPCR and western blot (WB) analysis of Cftr in primary chondrocytes pretreated with siNC or siCftr. Data are expressed as means ± SD. **b**-**c** Western blot analysis showing p-Akt, Caspase 3, and Bcl-2 expression in IL-1β-stimulated chondrocytes treated with roflumilast, and with or without si-Cftr. Data are expressed as means ± SD. Statistical analysis was performed using one-way ANOVA followed by Tukey’s post hoc test for comparisons among multiple groups. **d**-**e** TUNEL staining and flow cytometry assays for apoptosis in IL-1β-stimulated chondrocytes treated with roflumilast, and with or without si-Cftr. Data are expressed as means ± SD. Statistical analysis was performed using one-way ANOVA followed by Tukey’s post hoc test for comparisons among multiple groups. **d**-**e** Intracellular chloride concentrations measured by MQAE in IL-1β-stimulated chondrocytes treated with roflumilast, and with or without si-Cftr. Data are expressed as means ± SD. Statistical analysis was performed using one-way ANOVA followed by Tukey’s post hoc test for comparisons among multiple groups. *P < 0.05 was considered statistically significant

To further determine whether the anti-apoptotic effects of roflumilast were mediated through CFTR, we used CFTRinh-172 (a CFTR inhibitor) and si-Cftr in our experiments. The data indicated that inhibiting CFTR function with CFTRinh-172 (20 µM) or reducing CFTR expression with si-Cftr effectively reversed the protective effects of roflumilast, resulting in elevated expression of apoptotic proteins (Bax, Caspase 3) and reduced expression of p-Akt and Bcl-2 (Figs. 6a and b and 7b and c). This suggests that CFTR plays a crucial role in mediating the anti-apoptotic effects of roflumilast in chondrocytes. Furthermore, the TUNEL and flow cytometry assays indicated that the inhibition of chondrocyte apoptosis by roflumilast was negated by CFTR inhibition or reduced CFTR expression (Figs. 6c and d and 7d and e). Considering CFTR’s function as a chloride channel, we also measured intracellular chloride concentrations using MQAE. The results demonstrated that roflumilast mitigated IL-1β-induced chloride accumulation in chondrocytes, an effect that was reversed by CFTR inhibition or reduced expression (Figs. 6c and d and 7d and e). Overall, these findings indicate that CFTR is a key mediator of the anti-apoptotic effects of roflumilast on chondrocytes. These findings indicated that CFTR mediated roflumilast-induced chondroprotection through Akt/Bcl-2–dependent anti-apoptotic signaling.

To further determine whether the anti-apoptotic effects of roflumilast were mediated through CFTR, we used CFTRinh-172 (a CFTR inhibitor) and si-*Cftr* in our experiments. The data indicated that inhibiting CFTR function with CFTRinh-172 (20 µM) reversed the protective effects of roflumilast, resulting in significantly elevated expression of apoptotic proteins (Caspase 3) (*p* = 0.0084 vs. IL-1β + Rof group for Caspase 3) and significantly reduced expression of p-Akt/ Akt and Bcl-2 (*p* = 0.0250 vs. IL-1β + Rof group for p-Akt/ Akt; *p* = 0.0154 vs. IL-1β + Rof group for Bcl-2) (Figs. 6a and b and 7b and c). Similar effects were achieved after reducing CFTR expression using small interfering RNAs targeting *Cftr*. This suggests that CFTR plays a crucial role in mediating the anti-apoptotic effects of roflumilast in chondrocytes.

Furthermore, TUNEL and flow cytometry assays indicated that the inhibition of chondrocyte apoptosis by roflumilast was significantly negated by CFTR inhibition (Fig. 6c, d *p* = 0.0061, *p* < 0.001 IL-1β + Rof + inh-172 vs. IL-1β + Rof group) or reducing CFTR expression (Fig. 7d, e *p* = 0.0004, *p* < 0.0001 si*Cftr* IL-1β + Rof vs. siNC IL-1β + Rof group) (Figs. 6c and d and 7d and e). Considering CFTR’s function as a chloride channel, we also measured intracellular chloride concentrations by MQAE. The results demonstrated that roflumilast significantly mitigated IL-1β-induced chloride accumulation in chondrocytes, but an effect that was significantly reversed by CFTR inhibition (Fig. 6c, d *p* = 0.0076 IL-1β + Rof + inh-172 vs. IL-1β + Rof group)or reducing expression (Fig. 7d, e *p* = 0.0014, si*Cftr* IL-1β + Rof vs. siNC IL-1β + Rof group). Overall, these findings indicate that CFTR is a key mediator of the anti-apoptotic effects of roflumilast on chondrocytes. These findings indicated that CFTR mediated roflumilast-induced chondroprotection through Akt/Bcl-2–dependent anti-apoptotic signaling.

## Discussion

In this study, we evaluated the therapeutic potential of roflumilast in OA, focusing on its effects on ECM homeostasis, apoptosis, and the underlying signaling pathways in chondrocytes. Our principal findings are: (a) roflumilast reduced ECM degradation and chondrocyte apoptosis under inflammatory conditions; (b) it alleviated cartilage damage, subchondral bone remodeling, and synovial inflammation in vivo; and (c) these protective effects were mediated by CFTR activation and downstream anti-apoptotic signaling.

**Fig. 8:**
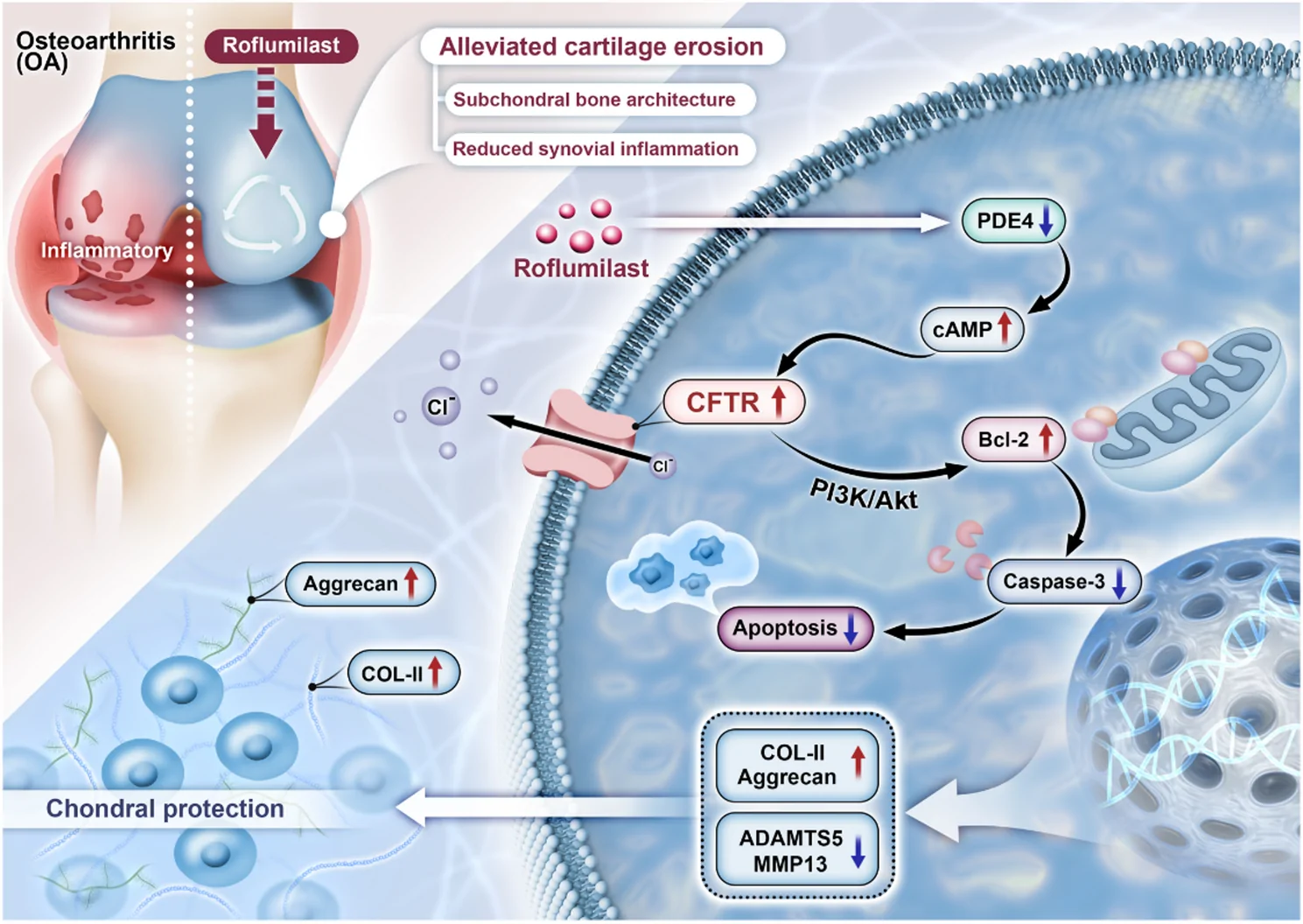
Schematic illustration of the proposed mechanism by which roflumilast attenuates osteoarthritis progression. Roflumilast exerts chondroprotective effects by regulating CFTR-dependent signaling in chondrocytes. Activation of CFTR further engages the PI3K/Akt/Bcl-2 signaling axis, thereby reducing chondrocyte apoptosis and ECM degradation under inflammatory stimulation. In vivo, these effects may contribute to reduced cartilage degeneration, subchondral bone remodeling, and synovial inflammation in DMM-induced osteoarthritis

Prior research has explored roflumilast’s impact in arthritic conditions, extending beyond its established roles in respiratory diseases. Notably, roflumilast has been shown to protect against collagen-induced arthritis (CIA) in mice, reducing joint inflammation and bone/cartilage erosion, and suppressing LPS-induced TNF production in vivo [23]. Furthermore, in fibroblast-like synoviocytes (FLS), a key cell type in rheumatoid arthritis (RA) pathogenesis, roflumilast effectively attenuated IL-18-induced oxidative stress, suppressed pro-inflammatory cytokines (IL-6, IL-8, TNF-α), chemokines (CCL5, CXCL9, CXCL10), and matrix-degrading enzymes (MMP-3, MMP-13), by inhibiting AP-1 and NF-κB activation [24]. These findings in RA-relevant cell types and animal models provide crucial context for understanding roflumilast’s therapeutic potential in joint diseases, including OA. (Fig. 8)

The observed improvement in locomotor performance of DMM mice treated with roflumilast, evidenced by enhanced gait parameters and increased spontaneous activity, suggests a potential alleviation of pain perception alongside structural and molecular chondroprotection. Roflumilast’s well-documented anti-inflammatory properties, achieved by elevating intracellular cAMP and suppressing pro-nociceptive mediators like TNF-α and IL-1β while upregulating anti-inflammatory cytokines [25, 26], likely contribute to this functional benefit. These inflammatory cytokines are known to sensitize joint nociceptors, playing a critical role in OA pain [27, 28]. By mitigating this inflammatory milieu, roflumilast could indirectly reduce nociceptor sensitization and activity, thereby alleviate pain and improve locomotion, consistent with analgesic effects observed for PDE4 inhibitors in various inflammatory pain models [29, 30]. While direct assessment of nociceptor presence or activity was beyond this study’s scope, the comprehensive anti-inflammatory actions of roflumilast provide a strong mechanistic basis for the observed functional improvements, indicating reduced pain perception as a likely contributing factor to the protective phenotype.

Phosphodiesterase 4 (PDE4) inhibitors are well-recognized for their anti-inflammatory properties and have been extensively investigated in various arthritic diseases. Tenor et al. (2002) demonstrated that human OA chondrocytes express PDE4, and its selective inhibition partially reduces IL-1β-induced nitric oxide (NO) generation, suggesting direct chondroprotective effects within the OA joint. Apremilast, another selective PDE4 inhibitor, has shown significant efficacy in RA and psoriatic arthritis (PsA). Studies indicate that apremilast inhibits spontaneous TNF-α production from human rheumatoid synovial cells and ameliorates experimental arthritis in murine models, notably without the adverse behavioral effects often associated with classical PDE4 inhibitors like rolipram [31]. Further research confirms that apremilast delays arthritis onset, reduces arthritis scores, and prevents bone erosion in CIA models, while also inhibiting the destructive and migratory ability of RA synovial fibroblasts (RASFs) to cartilages [32, 33]. Apremilast achieves these effects by elevating intracellular cAMP and modulating a network of pro-inflammatory mediators including TNF-α, IL-23, and IL-17 [33]. Recent reviews further highlight apremilast’s therapeutic mechanisms in OA, emphasizing its ability to suppress pro-inflammatory cytokines, protect chondrocytes, inhibit angiogenesis, and reduce oxidative stress [34]. While both roflumilast and apremilast are PDE4 inhibitors, our study uniquely identifies CFTR activation as a novel, specific downstream mediator of roflumilast’s anti-apoptotic effects in chondrocytes. This offers a distinct mechanistic insight within the broader context of PDE4 inhibition, suggesting a potentially unique or complementary therapeutic avenue for OA compared to other PDE4 inhibitors.

Chondrocyte apoptosis is a recognized contributor to OA pathogenesis and correlates with disease severity [35]. The PI3K/Akt pathway promotes cell survival through regulation of Bcl-2 family proteins, which govern mitochondrial membrane permeability and caspase activation [36, 37]. The balance between pro- and anti-apoptotic Bcl-2 members determines mitochondrial integrity and cell fate [38, 39]. Caspase-3, the key executioner caspase, induces DNA fragmentation by cleaving the inhibitor of caspase-activated DNase [40, 41]. Thus, suppressing caspase-3 activity is essential for maintaining chondrocyte viability. Given the reciprocal relationship between apoptosis and ECM degradation [9], targeting apoptosis has emerged as an attractive therapeutic approach for OA. Consistent with this concept, our in vivo data showed that roflumilast not only protected cartilage at the molecular and structural levels but also improved locomotor performance in DMM-induced OA mice, as reflected by enhanced gait parameters, increased spontaneous activity, reduced osteophyte formation, and lower synovitis scores. These findings indicate that roflumilast exerts protective effects across multiple levels of the joint microenvironment, translating into functional benefits.

Our study uniquely identifies CFTR activation as a novel mechanism for roflumilast’s chondroprotective effects in OA. CFTR, a membrane-bound ATP-binding cassette transporter, functions as a cAMP-regulated chloride channel activated by PKA-mediated phosphorylation [42, 43]. By inhibiting PDE4, roflumilast enhances intracellular cAMP and subsequently promotes CFTR activation. CFTR has been implicated in inflammation, autophagy, and cell survival [44–50] (Strubberg et al., 2018; Rubin, 2018; Fonseca et al., 2023; Erfinanda et al., 2022; Xia et al., 2016; Zhao et al., 2020; Zhang et al., 2018; Zeng et al., 2014), and cartilage abnormalities in CFTR-deficient mice highlight its importance in joint homeostasis [51]. Prior studies have confirmed CFTR expression in chondrocytes at both mRNA and protein levels, where it functions as a cAMP-dependent Cl⁻ channel [52]. Consistent with these findings, our analysis of human OA cartilage revealed markedly reduced CFTR expression in injured regions compared with paired uninjured sites, suggesting that CFTR downregulation is an intrinsic feature of human OA pathology. This observation provides translational support for the mechanistic link between CFTR deficiency and chondrocyte vulnerability. The interaction between CFTR and PI3K/Akt signaling has been observed in multiple pathological contexts [50, 53, 54]. For example, CFTR-deficient macrophages show impaired Akt phosphorylation and enhanced inflammatory responses [55], while in tumor cells CFTR promotes proliferation through PI3K/Akt signaling [50]. In our study, roflumilast-induced CFTR activation enhanced Akt phosphorylation and Bcl-2 expression while suppressing caspase-3 activation, thereby attenuating chondrocyte apoptosis. This mechanism likely underlies the preservation of ECM integrity and the delay of OA progression.

Several limitations should be acknowledged. First, detailed patient characteristics, including complete records of comorbidities and previous treatments, were not systematically collected during sample acquisition. Although the human cartilage samples were obtained from clinically diagnosed end-stage OA patients who underwent total knee replacement surgery, future studies with more comprehensive clinical information will be needed to further evaluate the relationship between patient characteristics and roflumilast-related molecular changes in OA cartilage. Second, the current in vivo experiments mainly evaluated the protective effect of roflumilast during early OA progression, and delayed treatment after the establishment of OA should be performed in future studies. Third, pain thresholds and nociceptor innervation/activity were not assessed, and whether the improved locomotor function is associated with reduced OA-related pain remains to be determined.

In conclusion, our data indicate that the chondroprotective effects of roflumilast are CFTR-dependent and mediated by the PI3K/Akt/Bcl-2 signaling axis. The loss of protection upon CFTR inhibition or knockdown further reinforces its central role. Moreover, the consistent downregulation of CFTR in human OA cartilage underscores its clinical relevance and highlights the therapeutic potential of pharmacological CFTR activation. Future studies should define the optimal dosage and delivery route for intra-articular CFTR modulation and further clarify the molecular interplay between CFTR and apoptotic signaling. Combining roflumilast with cartilage-regenerative strategies may represent a promising avenue for disease modification in OA.

## Supplementary Information

Supplementary Material 1.

## Data Availability

All raw RNA-seq data generated in this study have been deposited in the NCBI SRA under BioProject accession number PRJNA1246960 and are accessible at: [https://www.ncbi.nlm.nih.gov/sra/PRJNA1246960](https:/www.ncbi.nlm.nih.gov/sra/PRJNA1246960) . Any additional information required to reanalyze the data reported in this paper is available from the lead contact upon request.
